# Quantification of the Frequency and Multiplicity of Infection of Respiratory- and Lymph Node–Resident Dendritic Cells During Influenza Virus Infection

**DOI:** 10.1371/journal.pone.0012902

**Published:** 2010-09-23

**Authors:** Rebecca VanOosten Anderson, Jodi McGill, Kevin L. Legge

**Affiliations:** Department of Pathology, Interdisciplinary Graduate Program in Immunology, University of Iowa, Iowa City, Iowa, United States of America; University of California Los Angeles, United States of America

## Abstract

**Background:**

Previous studies have demonstrated that DC differentially regulate influenza A virus (IAV)–specific CD8 T cell responses *in vivo* during high and low dose IAV infections. Furthermore, *in vitro* infection of DC with IAV at low versus high multiplicities of infection (MOI) results in altered cytokine production and a reduced ability to prime naïve CD8 T cell responses. Flow cytometric detection of IAV proteins within DC, a commonly used method for detection of cellular IAV infection, does not distinguish between the direct infection of these cells or their uptake of viral proteins from dying epithelial cells.

**Methods/Principal Findings:**

We have developed a novel, sensitive, single-cell RT-PCR–based approach to assess the infection of respiratory DC (rDC) and lymph node (LN)-resident DC (LNDC) following high and low dose IAV infections. Our results show that, while a fraction of both rDC and LNDC contain viral mRNA following IAV infection, there is little correlation between the percentage of rDC containing viral mRNA and the initial IAV inoculum dose. Instead, increasing IAV inoculums correlate with augmented rDC MOI.

**Conclusion/Significance:**

Together, our results demonstrate a novel and sensitive method for the detection of direct IAV infection at the single-cell level and suggest that the previously described ability of DC to differentially regulate IAV-specific T cell responses during high and low dose IAV infections could relate to the MOI of rDC within the LN rather than the percentage of rDC infected.

## Introduction

Following influenza A virus (IAV) infection, respiratory dendritic cells (rDC) mature, upregulate costimulatory molecules, and migrate to the regional lymph nodes (LN) where they participate in the induction of virus-specific CD8 T cell responses [1], [2], [3]. Importantly, the signals provided to naïve CD8 T cells through DC interactions and the corresponding cytokine environment within the LN are known to shape the type of T cell response generated. For example, IL-12p70 has been shown to promote increased CD8 T cell survival, CD25 upregulation and cytolytic activity [4], [5], [6]. Because of their essential role in regulating the induction of adaptive immune responses, it is not surprising that migratory rDC and LNDC have been implicated as key players in governing the phenotype and magnitude of adaptive immune responses following lethal vs. sublethal dose IAV infections.

Previous studies have shown that *in vitro* infection of bone-marrow derived DC [7], [8], [9] or isolated murine respiratory DC [10] with IAV can influence subsequent DC cytokine production and priming of virus-specific CD8 T cell responses. Interestingly, while IAV infection of BMDC at low multiplicities of infection (MOI) resulted in enhanced CD8 T cell responses following *in vitro* stimulation, high MOI resulted in blunted CD8 T cell responses, increased IL-12p40 production [8] and increased production of the anti-inflammatory cytokines IL-10 and TGF-beta [9]. Additionally IAV infection of pulmonary rDC at high, but not low MOI, resulted in increased production of IL-6, MIP-1α and IFNα by certain DC subsets [10].

Similar to these in vitro studies, we have demonstrated that rDC and LNDC produce an increased concentration of IL-12p40 during a high-dose IAV infection setting that results in LNDC-mediated killing of CD8 T cells, a blunted virus-specific CD8 T cell response in the lungs, and ultimately, a lethal outcome [11]. Further, we and others have demonstrated that IAV protein expression could be detected by flow cytometry in rDC that have migrated from the lungs to the LN following both high and low dose infections, but not on LNDC in either infection setting [10], [11], [12]. Importantly, the frequency and MFI of those cells expressing viral nucleocapsid protein (NP), hemagglutinin (HA) and nonstructural-1 protein (NS-1) is increased in mice receiving lethal doses of IAV relative to those receiving sublethal doses of infection [10], [11]. These results suggested that the increased viral infection of migratory rDC in lethal infection settings may play an important role in regulating the ensuing adaptive CD8 T cell response and ultimately, disease outcome.

The flow cytometry-based method of detection of IAV proteins in DC is particularly valuable due to the high-throughput nature of the analysis and the ability to examine very small populations of infected cells. However, flow cytometry lacks sufficient sensitivity to quantify viral load or discern infected cells from those that have taken up viral antigens. Additionally, if the migratory rDC are indeed directly infected during low and high dose settings, it is impossible to quantify the amount of virus infecting each cell. Given the hypothesized importance of MOI in regulating DC function and ability to promote robust CD8 T cell responses [7], [8], [9], [10], we assessed DC infection by a more sensitive and quantitative approach. PCR for viral mRNA has been previously used to detect viral infection on bulk tissues such as the mediastinal lymph nodes and lungs [13], [14], [15]. Therefore we used single cell RT-PCR [16], [17] to detect the presence of viral mRNA, particularly spliced mRNA from IAV genes, in individual DC. This technique may more accurately define direct IAV infection as the spliced mRNA should have a short half-life and only be expressed during active viral replication. Further, by monitoring the PCR cycle threshold during the RT-PCR analysis, one should be able to estimate the relative viral load of the infected cells. Our results show that a fraction of rDC and LNDC contain viral mRNA following both high and low dose IAV infections, and that the frequency of IAV mRNA+ DC present within the LN does not correlate with the increasing viral inoculum. Instead, we demonstrate that relative mRNA load per cell correlates with increasing initial viral inoculum, particularly within the migratory rDC subset. Together, these results suggest that *in vivo* rDC infection and hence, the magnitude of adaptive responses and outcome of disease, may depend on the multiplicity of infection (MOI) of migratory rDC.

## Results

### Comparison of IAV protein and mRNA expression in *in vitro* IAV-infected XS106 DC

**Figure 1:**
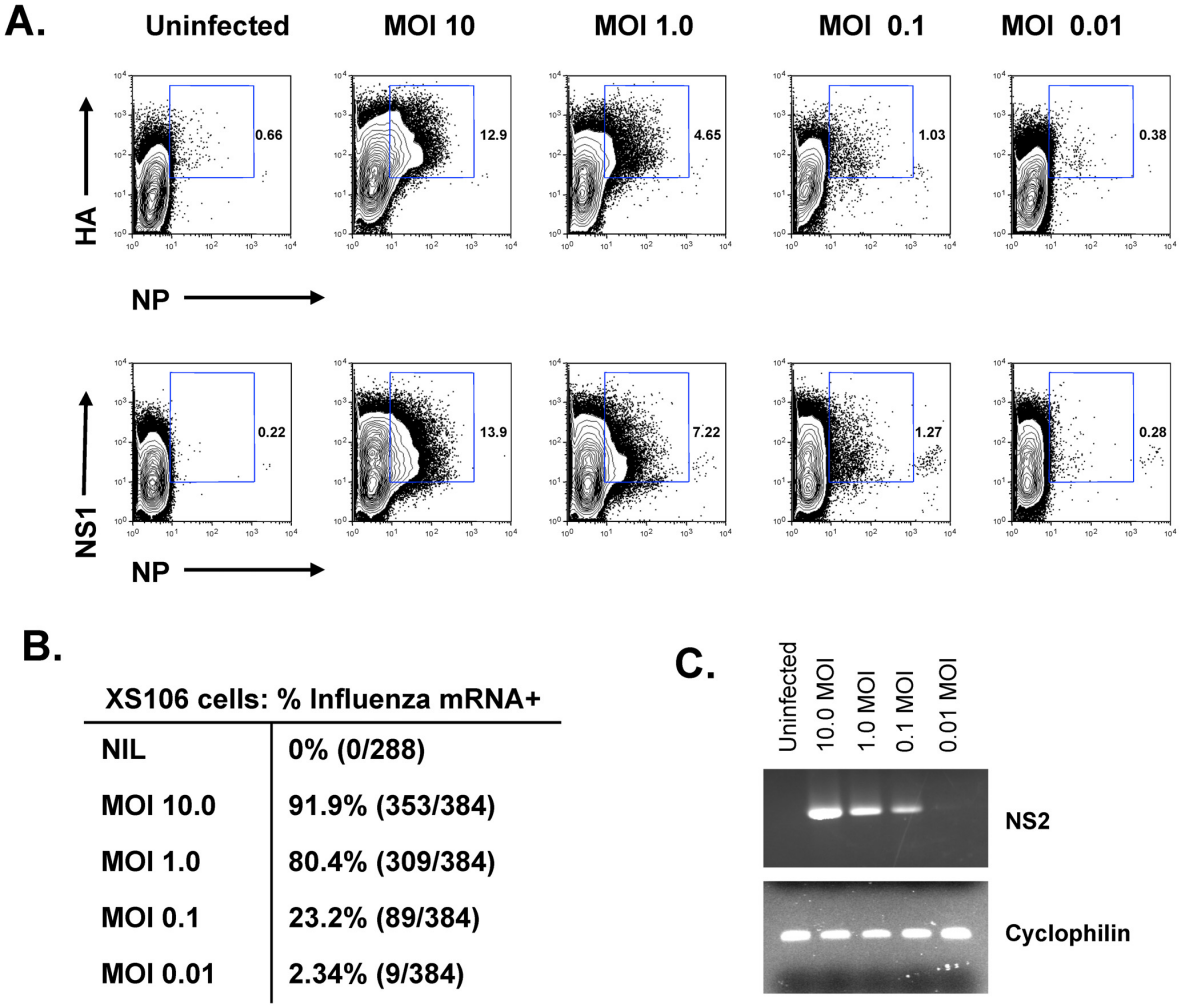
Single cell RT-PCR and flow cytometry following increasing MOI in the dendritic-like cell line XS106. XS106 cells were infected with 0, 0.01, 0.1, 1 and 10 MOI of IAV. 18 hours p.i., cells were harvested and analyzed by flow cytometry (A) for the IAV proteins hemagglutinin (HA) and nucleocapsid protein (NP) (top panels) or HA and nonstructural-1 (NS-1) (bottom panels) or (B and C) single-cell PCR for the viral nonstructural protein 2 (NS-2) mRNA. Data are representative of 2 separate experiments. Cyclophilin normalized intensities in C; 0.865: 10 MOI; 0.640: 1MOI; 0.388: 0.1MOI; 0.068: 0.01MOI.

In order to compare single-cell RT-PCR analysis to flow cytometry analysis for IAV proteins, we initiated experiments using the DC-like cell line XS106. XS106 cells were infected *in vitro* with increasing MOI of the IAV. At 18 hours p.i., the cells were harvested and analyzed by flow cytometry for IAV proteins (Fig 1A) or by single-cell RT-PCR for presence of IAV NS2 mRNA (Fig 1B and C). Not unexpectedly, the frequency of XS106 cells with detectable IAV HA and NP proteins (Fig 1A, top panels) or influenza NP and NS1 proteins (Fig 1A, bottom panels) by flow cytometry was proportional to the MOI, with approximately 13% of cells expressing IAV proteins following a 10.0 MOI infection and approximately 1% of cells expressing IAV proteins following a 0.1 MOI infection (Fig 1A). In contrast, when cells were analyzed by single cell RT-PCR analysis for influenza NS2 mRNA, we observed that over 90% of the XS106 cells expressed viral mRNA following a 10 MOI infection and at least 2% of the cells still expressed viral mRNA even after a 0.01 MOI infection (Fig 1B). Importantly, the primers for detecting viral mRNA expression were specific and did not display cross-reactivity, as we observed no amplification of bands in uninfected cells. In addition to the correlation between decreasing frequencies of infected cells and decreasing MOI, we also observed a correlation in the band intensity and MOI (Fig 1C). While cells infected at a 0.01 MOI showed only a faint band for NS2 mRNA expression (0.068 intensity relative to cyclophilin), those infected at a 10 MOI showed a more intense band (0.865 intensity relative to cyclophilin), suggesting increased viral loads in cells infected at high MOI relative to those infected at low MOI. Together, these results demonstrate that single cell RT-PCR for IAV mRNA expression is more sensitive than results obtained using flow cytometry and that this method can be successfully applied to detect relative viral loads between infected cells.

### Infection of migratory rDC and LNDC following high and low dose IAV infections as measured by RT-PCR

**Figure 2:**
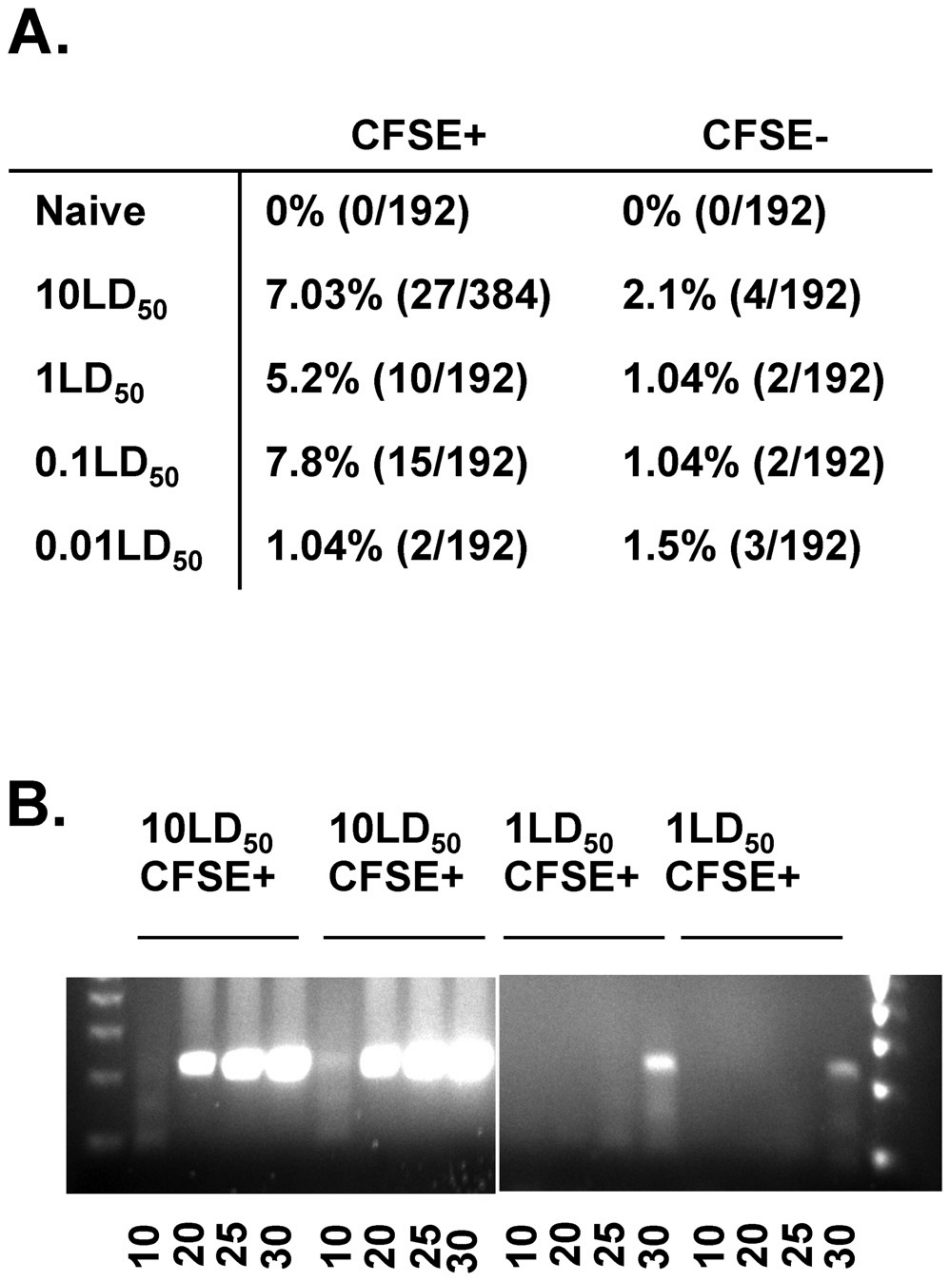
Single cell RT-PCR following increasing inoculum doses. BALB/c mice were treated i.n. with CFSE. 6 hours later, groups of mice (20 mice/group) were left uninfected (Naïve) or infected with the indicated dose of IAV. At 18 hours p.i., draining LN (20 mice/group) were pooled and migratory rDC (CFSE^+^CD11c^+^MHC II^+^) and LNDC (CFSE^neg^CD11c^+^MHC II^+^) were single-cell FACS sorted into 96 well plates and analyzed by single-cell RT-PCR for expression of viral NS-2 mRNA as described in Materials and Methods. (A) Data represent the frequency of NS-2 IAV mRNA^+^ cells/total cells analyzed. (B) cDNA from positive samples in (A) was again amplified by nested PCR for NS2. Indicated is the cycle at which the PCR reaction was stopped during the second nested PCR reaction (i.e. the amplification with the NS2C:NS3 primers).

We next utilized the single-cell PCR methodology to examine infection of migratory rDC and LNDC in high and low dose *in vivo* IAV infections. To this end, BALB/c mice were treated intranasally (i.n.) with CFSE to specifically label the DC present in the lungs. Six hours later, the mice were infected with increasing inoculums of IAV and at 18 hours post infection (p.i.), CFSE^+^CD11c^+^MHC-II^+^ migratory rDC and CFSE^neg^CD11c^+^MHC-II^+^ LNDC were FACS purified from the pooled draining LN and analyzed by RT-PCR for IAV NS2 mRNA expression. In agreement with previous results obtained by flow cytometry for IAV proteins [10], [11], we observed IAV mRNA in migratory CFSE^+^CD11c^+^MHC-II^+^ rDC at both high and low inoculum doses (Fig 2A). Interestingly, however, while previous flow cytometry results have suggested a correlation between the IAV inoculation dose and the frequency of rDC that expressed IAV proteins, we instead observed that a similar frequency (5–7%) of CFSE^+^ rDC were infected at the 10, 1 and 0.1 LD_50_ inoculum doses by RT-PCR analysis. However, at a very low inoculum dose of virus (i.e. a 0.01 LD_50_ dose), there was a decreased level of infection of CFSE^+^ rDC, with only 1% of rDC expressing viral mRNA (Figure 2A). In further contrast with the results obtained using flow cytometry for viral proteins suggesting no infection of LNDC [11], the more sensitive single-cell RT-PCR approach revealed that a small fraction of CFSE^neg^ LNDC were IAV NS2 mRNA^+^, even at very low inoculum doses. Infection of LNDC did not appear to correlate with inoculum dose, as ∼1–2% of LNDC are infected in both lethal (10 and 1 LD_50_ doses) and sublethal (0.1 and 0.01 LD_50_ doses) infection settings (Fig 2A). Together, these results suggest that while migratory rDC and LNDC are infected during a primary IAV infection, there is little correlation between the frequency of infection in either cell type and the initial inoculum load.

### Increased MOI in migratory rDC following high vs. low dose IAV infections

Given the results of Oh et al. [9] suggesting that increasing MOI can have adverse effects on the ability of DC to promote robust adaptive CD8 T cell responses, we next utilized the single-cell RT-PCR approach to compare the MOI of DC from low and high dose IAV infections. To this end, we used increasing cycle numbers to distinguish the level of IAV infection in migratory CFSE^+^ rDC from 10 LD_50_ vs. 1 LD_50_ infections. Strikingly, while viral NS2 mRNA was detectable in rDC purified from a 10 LD_50_ dose infection setting after as few as 10 cycles (Fig 2C, left), it took 30 cycles to detect viral mRNA in rDC purified from a 1 LD_50_ dose infection setting (Fig 2C, right). Overall, these results suggest that rDC from high dose infection settings may have been infected with more IAV virions than rDC from lower dose infections. These results further suggest that an increase in influenza MOI in rDC following high vs. low dose infections may be key in regulating the magnitude of the adaptive CD8 T cell response [7], [8], [9], [11].

## Discussion

Here, we have developed a novel, sensitive, *in vivo* single-cell RT-PCR approach for directly analyzing IAV infection of rDC and LNDC following high and low dose infection. The RT-PCR method of analysis offers several advantages over the previously utilized flow cytometry approach. While the flow cytometry based method allows one to detect the presence of IAV proteins, it lacks the ability to discern between direct infection of DC and the presence of IAV protein as a result of phagocytosing infected epithelial cells. Given previously published results demonstrating the significant effects of direct IAV infection on DC cytokine production and the ability to prime adaptive T cell responses *in vitro*, it is essential to understand the relationship between IAV infection and DC function *in vivo*. Our newly described method of single-cell RT-PCR detects spliced mRNA from the IAV NS2 gene, in individual DC. This technique more accurately defines direct IAV infection as the spliced mRNA is expected to have a short half-life, is only expressed during active viral replication, and is present a much reduced levels compared to the IAV NP and HA proteins used for flow cytometry-based detection [18]. Paired with complimentary analysis for cytokine and chemokine mRNA, the single-cell RT-PCR approach may prove very powerful for studying the results of direct infection on DC function and the resulting adaptive immune response *in vivo*.

As a further advantage over the current flow cytometry based method of IAV protein detection, the single-cell RT-PCR approach also offers increased sensitivity and the ability to quantitate the amount of virus load per cell. Previously utilized flow cytometry analysis has suggested infection of rDC *in vivo* following high dose (i.e. 1 and 10 LD_50_), but not low dose (i.e. 0.1 LD_50_) IAV infection [11]. Further, the flow cytometry based analysis did not detect the presence of IAV infection in LNDC [11]. In contrast, our results using the more sensitive single-cell RT-PCR based method demonstrate that IAV mRNA is present in both migratory rDC and LNDC *in vivo* following both high and low dose infection. We further demonstrate that, in direct contrast to those results obtained using the flow cytometry based method of detection [11], a similar frequency of rDC (5–7%) and LNDC (1–2%) are infected *in vivo* following either high or low doses of infection. The single-cell RT-PCR approach also allows one to quantitate the viral load per cell. We demonstrate here that the MOI of migratory rDC differs significantly with respect to the initial viral inoculum (Figure 2B). Interestingly, at high doses of infection, results from the flow cytometry based method correlate well with the results from the RT-PCR based method: increasing viral inoculum results in increased MFI by flow cytometry (Figure 1A and [11]) or increased MOI by RT-PCR (Figure 1C and 2A). However, with lower doses of infection (i.e. 0.1 LD_50_ or less), the MFI by flow cytometry is below the limit of detection while the more sensitive RT-PCR method still detects the presence of viral mRNA, indicative of DC infection, and a difference in the quantity of viral load per cell. Together, these results suggest that while the flow cytometry based method is invaluable for its high-throughput nature and ability to study very rare populations of cells directly *ex vivo*, our novel single-cell PCR based method is more accurate and sensitive for the detection and quantification of direct viral infection and will prove essential for determining the effects of direct infection on DC function *in vivo*.

At this time, the source of IAV mRNA in CFSE^neg^ LNDC remains unclear. We have previously demonstrated that the i.n. CFSE labeling technique labels all cell types present in the respiratory tract, and results in little to no non-specific labeling of cells in the draining LN [1]. Therefore, it is unlikely that the CFSE^neg^ DC are unlabeled cells that traveled from the lungs to the LN. It is possible that LNDC obtained IAV mRNA through the phagocytosis of infected rDC that migrated from the lungs to the LN following infection. However, given the low frequency of IAV mRNA positive cells in the LN, and the technical difficulty associated with sorting very rare populations of DC, this could also be an artifact resulting from cell sorting contamination. Future studies should be aimed at confirming if direct infection of LNDC occurs *in vivo* following high and low dose infections and, if so, how this alters the priming of adaptive immunity therein.

It is known that the magnitude of the adaptive response shares an inverse correlation with the initial viral inoculum [11]. Direct infection of DC with low MOI *in vitro* results in significant production of pro-inflammatory cytokines and priming of a robust CD8 T cell response, while infection of DC with a high MOI results in increased production of anti-inflammatory cytokines such as IL-10 and TGF-beta, and a reduced ability to prime CD8 T cell immunity [9]. Together these results along with the results herein demonstrating that increasing doses of IAV result in increased MOI of migratory rDC suggest that the magnitude of rDC infection may be a key feature in the regulation of adaptive CD8 T cell responses following lethal vs. sublethal IAV infections in mice.

## Materials and Methods

### Ethics Statement

Experiments were conducted according to federal and institutional guidelines and all animal experiments were approved by the University of Iowa Animal Care and Use Committee (Protocol #s: 0611238, 0911255).

### Mice

Female BALB/c mice were purchased from the National Cancer Institute (Frederick, MD) and used for experiments at 6–8 weeks of age. Mice were allowed access to food and water *ab libitum*. Mice were housed and maintained under specific-pathogen free conditions in the animal care facility at the University of Iowa. All animals were handled in strict accordance with good animal practice as defined by the relevant national and/or local animal welfare bodies, and all animal work was approved by the University of Iowa Animal Care and Use Committee.

### Tissue Culture

NS46/47 fibroblast cell lines and XS106 dendritic cell lines were a kind gift from Dr. Akira Takashima (University of Toledo) [19], [20], [21]. NS46/47 fibroblast feeder cell lines were grown in complete RPMI and the supernatant was collected and filter sterilized. XS106 A/J derived dendritic cell line was maintained in complete RPMI containing 5% sterile NS46/47 cell supernatant and 0.5 ng/ml mouse recombinant GM-CSF.

### Intranasal CFSE administration

Intranasal CFSE administration was performed 6 hours prior to influenza virus infection. Mice were anesthetized by isofluorane inhalation and administered 50 µl of 8 mM CFSE diluted in Iscove's DMEM as previously described [1].

### Influenza Virus Infection

Viruses were grown in 10 day old embryonated hen eggs, snap frozen and stored at −80°C as previously described [11]. XS106 cells were washed and suspended in serum-free RPMI. Mouse-adapted A/JAPAN/305/57 (H2N2) was then added to the culture and incubated to 30 minutes on ice, followed by 30 minutes at 37°C. Following infection, the cells were washed, returned to complete RPMI and cultured at 37°C for 18 hours prior to flow cytometry staining or single-cell PCR. Mice were anesthetized by isofluorane inhalation and infected i.n. with either a 10 LD_50_ (3.67×10^6^ EIU) (lethal dose, 100% lethality), 1 LD_50_ (3.67×10^5^ EIU) (lethal dose, ∼50% lethality), 0.1 LD_50_ (3.67×10^4^ EIU) (sublethal dose, ∼5% lethality) or 0.01 LD_50_ (3.67×10^3^ EIU) (sublethal dose, <1% lethality) dose of mouse-adapted A/JAPAN/305/57 (H2N2) in 50 µl of Iscove's media.

### Staining for Influenza Viral Antigen

XS106 A/J derived dendritic cell line cells were infected and harvested 18 hours p.i. Cells were fixed in BD FACS Lysing solution (BD Biosciences), permeabilized with 0.5% saponin and stained with antibodies to influenza Nucleoprotein (NP) antigen (clone H16, kindly provided by Walter Gerhardt, Wistar Institute), influenza hemagglutinin antigen (clone Fc125) or non-structural protein 1 (NS-1) antigen (clone 1A7, kindly provided by Jonathan Yewdell, NIAID) for 30 minutes at 4°C. All flow cytometry data was acquired on a BD FACS Calibur (BD Immunocytometry Systems) in CellQuest (BD) and analyzed using FlowJo software (TreeStar, Ashland, OR).

### Preparation of Lymph Node (LN) Dendritic Cells for single-cell RT-PCR

CFSE-labeled, IAV infected mice were harvested 18 hours p.i. Draining mediastinal and peribronchial LN were harvested, pooled and single cell suspensions were prepared. Cells were labeled with rat anti-mouse IA/IE (M5/114.15.2) and hamster anti-mouse CD11c (HL3) (both purchased from Becton Dickinson) for 30 minutes at 4°C. CD11c^+^MHC II^+^ cells were first sorted for bulk yield (Figure S1A), then single cells were subsequently sorted into 96 well plates based on CFSE positivity (Figure S1B). Sorting was performed on a BD FACS Diva (BD Immunocytometry Systems) that is equipped with three excitation sources: an Argon ion laser at 475.9 nm, 488 nm or 514.5 nm, a dye laser at 600 nm and a Krypton laser at 337.5–356.4 nm (UV) or 406.7 nM (violet); and has the ability to run 11 channels plus time.

### Single-Cell RT-PCR

The protocol for single cell RT-PCR was modified from [16], [17]. Single cells were sorted into a premix detergent containing OligoDT (Integrated DNA Technologies), 10% NP-40 (Sigma), 0.1 M DTT (Invitrogen) and rRNase OUT (Invitrogen), then stored at −80°C until use. cDNA synthesis was performed using the Reverse Transcription System (Promega) according to manufacturers instructions.

The following primers were ordered from Integrated DNA Technologies for PCR: Cyclophilin 1 Forward: GTGGTCTTTGGGAAGGTGAA; Cyclophilin 2 Reverse: TTACAGGACATTGCGAGCAG; Cyclophilin A Forward: GGCCGATGACGAGCCC; NS2A Forward: GATCCCAACACTGTGTCAAGC; NS5 Reverse: CGAGAAAGTTCTTATCTCTTGTTCC; NS2C Forward: CACTGTGTCAAGCTTTCAGGAC; NS3 Reverse: CTTCTTCAATCAGCCATCTTATTTC. All PCR amplifications used Red Hot Taq (AbGene), dNTPs (Invitrogen), 10X PCR butter and MgCl_2_ (ThermoScientific). Prior to amplification, all cDNA was diluted 1∶5. Amplification of Cyclophilin was performed by nested PCR as follows: round 1 using Cyclophilin 1 Forward and Cyclophilin 2 Reverse for 40 cycles (94°C for 30 seconds, 52.4°C for 30 seconds, 72°C for 2 minutes); round 2 using Cyclophilin A Forward and Cyclophilin 2 Reverse for 30 cycles (94°C for 30 seconds, 52.4°C for 30 seconds, 72°C for 2 minutes). Amplification of influenza NS2 mRNA was performed by nested PCR as follows: round 1 using NS2A Forward and NS5 Reverse for 40 cycles (94°C for 30 seconds, 59°C for 30 seconds, 72°C for 2 minutes); round 2 using NS2C Forward and NS3 Reverse for 45 cycles (94°C for 30 seconds, 57°C for 30 seconds, 72°C for 2 minutes). Samples were then run on a 1.5% Agarose gel. All positive samples were confirmed by a second PCR reaction from the remaining cDNA.

## Supporting Information

Figure S1 Single-cell sorting scheme. Single-cell sorting was performed in two steps. (A) An initial dendritic cell enrichment sort was performed on draining lymph node cells, enriching for CD11c^+^MHCII^+^ cells. Enrichment increased DCs from 0.93% (A) to 45.1% (B). (B) The enriched population was then sorted into 96 well plates containing lysis buffer based on CD11c^+^MHCII^+^ expression and divided into two populations, CFSE^+^ and CFSE^neg^.
(1.55 MB TIF)
