# Supplementary figures and images for: Quantification of the Frequency and Multiplicity of Infection of Respiratory- and Lymph Node–Resident Dendritic Cells During Influenza Virus Infection
Source: PLoS One. 2010 Sep 23;5(9):e12902. doi: 10.1371/journal.pone.0012902 (PMC2944834; doi:10.1371/journal.pone.0012902)

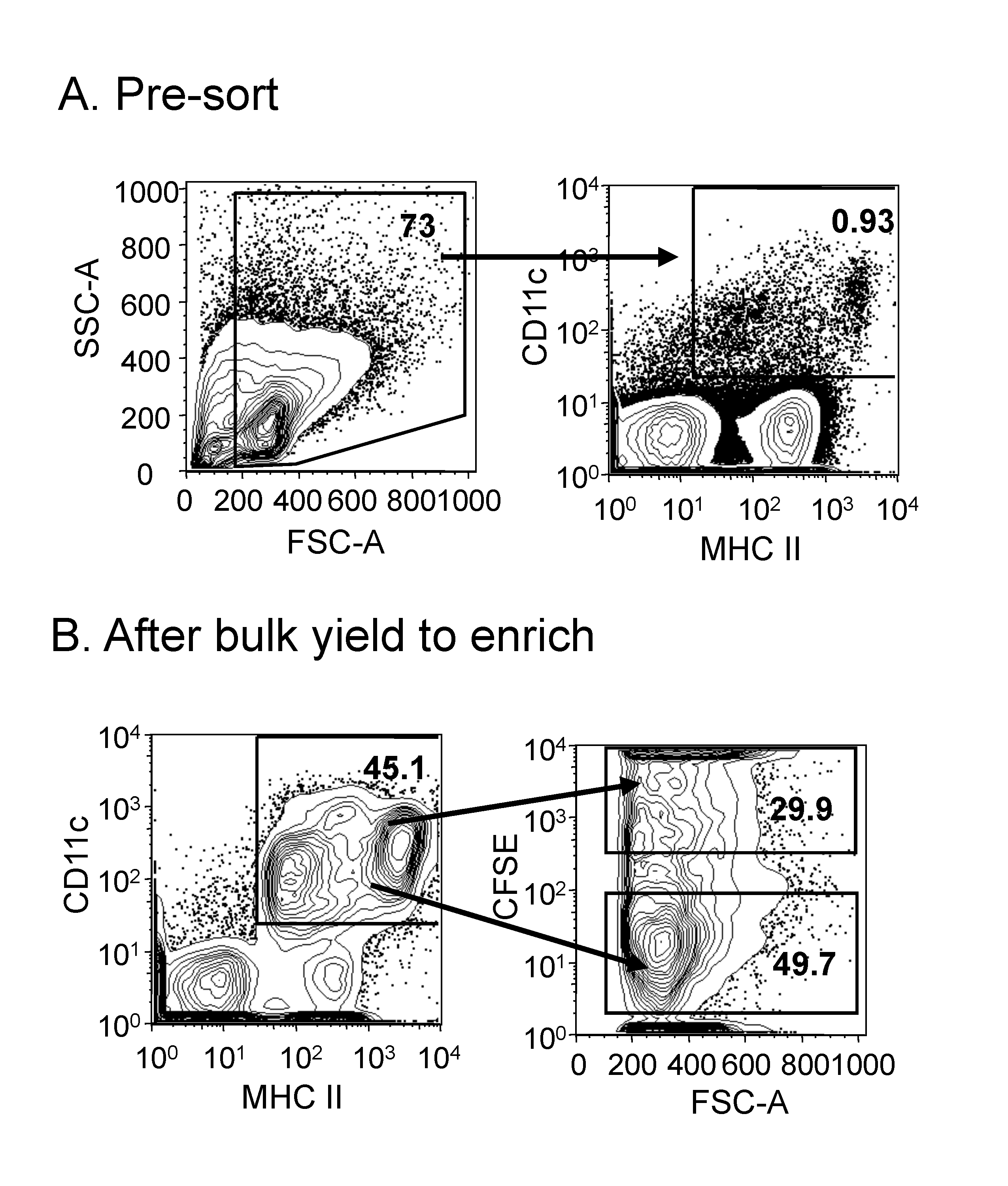

Supplement: Figure S1 — Single-cell sorting scheme. Single-cell sorting was performed in two steps. (A) An initial dendritic cell enrichment sort was performed on draining lymph node cells, enriching for CD11c+MHCII+ cells. Enrichment increased DCs from 0.93% (A) to 45.1% (B). (B) The enriched population was then sorted into 96 well plates containing lysis buffer based on CD11c+MHCII+ expression and divided into two populations, CFSE+ and CFSEneg. (1.55 MB TIF) [file pone.0012902.s001.tif]
